# Quantitative Detection Method for Surface Angled Cracks Based on Laser Ultrasonic Full-Field Scanning Data

**DOI:** 10.3390/s24237519

**Published:** 2024-11-25

**Authors:** Chenwei Wang, Rui Han, Yihui Zhang, Yuzhong Wang, Yanyang Zi, Jiyuan Zhao

**Affiliations:** 1 State Key Laboratory for Manufacturing Systems Engineering, Xi’an Jiaotong University, Xi’an 710049, China; iwangchenwei@163.com (C.W.);; 2 School of Automation, Beijing Information Science and Technology University, Beijing 100192, China

**Keywords:** surface angled crack, full-field scanning data, laser ultrasonic, quantitative detection method

## Abstract

Surface angled cracks on critical components in high-speed machinery can lead to fractures under stress and pressure, posing a significant threat to the operational safety of equipment. To detect surface angled cracks on critical components, this paper proposes a “Quantitative Detection Method for Surface Angled Cracks Based on Full-field Scanning Data”. By analyzing different ultrasonic signals in the full-field scanning data from laser ultrasonics, the width, angle, and length of surface angled cracks can be determined. This study investigates the propagation behavior of ultrasonic waves and their interaction with surface angled cracks through theoretical calculations. The crack width is solved by analyzing the distribution of Rayleigh waves in the full-field scanning data. This paper also discusses the differences in ultrasonic wave propagation between near-field and far-field detection and identifies the critical point between these regions. Different computational methods are employed to calculate the inclination angle and the crack endpoint at various scan positions. Four sets of experiments were conducted to validate the proposed method, with results showing that the errors in determining the width, angle, and length of the surface angled cracks were all within 5%. This confirms the feasibility of the method for detecting surface angled cracks. The quantitative detection of surface angled cracks on critical components using this method allows for a comprehensive assessment of the component’s condition, aiding in the prediction of service life and the mitigation of operational risks. This method shows promising application potential in areas such as aircraft engine blade inspection and gear inspection.

## 1. Introduction

In high-speed machinery, components often operate under harsh conditions such as a high temperature, high pressure, and high-speed rotation. Once small surface defects occur, they tend to propagate into the component due to the combined effects of stress and pressure, leading to crack defects and eventually causing fractures, which seriously threaten the safety of equipment operation [1,2,3]. Cracks caused by surface defects typically form at an angle to the surface due to the pressure applied. Conventional methods such as magnetic particle inspection [4], penetrant testing [5], and optical inspection [6] can only provide information about the crack width, but cannot determine the crack’s inclination angle or length. Traditional ultrasonic testing [7], with its low frequency, is not sensitive to small defects and poses challenges in deploying probes on complex structures. Additionally, radiographic inspection [8] and other methods are not sensitive to cracks and have low efficiency.

For surface angled cracks that propagate from surface defects into the interior, the focus of detection lies in determining the crack’s width, inclination angle and length. Given the difficulty of deploying sensors on complex structures, a non-contact, high-precision, and high-efficiency nondestructive testing (NDT) technique is required, and laser ultrasonic technology meets these requirements. Laser ultrasonics [9] uses a pulsed laser to generate ultrasonic waves in the test specimen and detects the propagation of the waves using laser beams. This technique combines the high precision of ultrasonic testing with the non-contact advantages of optical inspection. It features high sensitivity, broad bandwidth, and is suitable not only for surface defect detection [10,11], but also for internal defect detection [12,13,14] using longitudinal waves, offering promising applications in NDT.

Currently, several research institutions have conducted studies on detecting surface angled cracks using laser ultrasonics. Edwards et al. [15] investigates surface cracks using indicators such as ultrasonic transmittance, reflectance, and time delays, but it is not suitable for large cracks with a high aspect ratio. Zeng et al. [16] analyzes ultrasonic signals from cracks with different inclination angles in the time domain and calculates the crack’s inclination angle using a high signal-to-noise ratio (SNR) signal, but this does not provide a method to determine the crack length. Edwards et al. [17] discusses the characteristics of ultrasonic signals at different detection positions when detecting surface angled cracks and separately considers near-field and far-field detection. Lamb waves are used to analyze small-angled cracks in near-field detection, while in far-field detection, the angled crack is treated as a wedge, and body waves are used to determine the inclination angle. However, this study does not provide methods to calculate the crack length and width. Ni et al. [18] utilizes a finite element simulation and adopts a special detection method by directing the laser into the crack and using the propagation of ultrasonic waves to infer the crack inclination angle. While the simulation results meet expectations, the method is difficult to implement in practice due to issues with the laser spot size and focusing. Wang et al. [19] solves for the crack’s inclination angle and length by analyzing changes in the modes of transmitted waves. However, this method is based on the analysis of transmitted waves, and as the crack length increases, the attenuation of ultrasonic waves along the crack path increases, limiting the method’s applicability to shallow cracks rather than deep cracks.

In summary, current research on surface angled cracks lacks a universal, multidimensional method for evaluating cracks. Most existing studies employ single methods to solve for specific parameters of surface angled cracks, each of which has various limitations. This paper proposes a “Quantitative Detection Method for Surface Angled Cracks Based on Full-field Scanning Data”, and its effectiveness is validated through geometric derivation and experiments. By using this method for the quantitative detection of surface angled cracks on critical components, a comprehensive assessment can be achieved, aiding in the estimation of component service life and the proactive mitigation of operational risks.

## 2. The Establishment of Detection Method

In defect detection using laser ultrasonic technology, there are concepts of near-field and far-field detection. Near-field detection targets defects that are relatively close to the scanning position and whose shapes cannot be ignored, while in far-field detection, the defect shape is generally disregarded, focusing mainly on defect localization. For surface angled cracks with an unknown inclination direction and length, this paper proposes a “full-field scanning” method: without prior knowledge of the crack’s inclination direction, a long-distance linear B scan is performed to capture as much of the ultrasonic signal from the surface angled crack as possible, which is referred to as full-field scanning data. The full-field scan data include both near-field and far-field detection data, as well as data from cases where the generation and receiving points are located on opposite sides of the crack.

The detection of surface angled cracks requires evaluation from multiple dimensions. The core indicators include the crack’s opening width, inclination angle, and length. The full-field scanning data include signals from multiple scanning positions, revealing patterns of waveform changes at different scanning positions, which can be used to calculate the opening width, inclination angle, and length of the surface angled crack.

**Figure 1:**
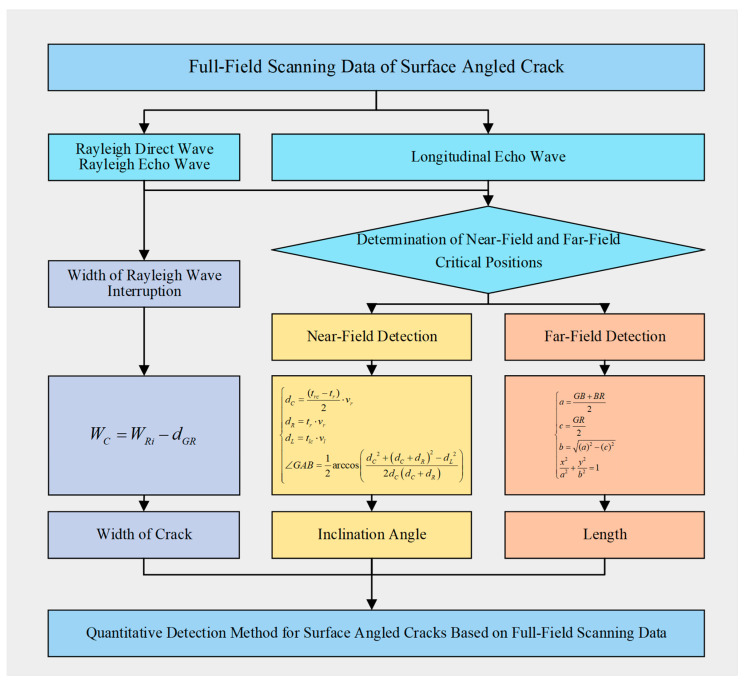
The core concept of Quantitative Detection Method for Surface Angled Cracks Based on Full-Field Scanning Data.

The core concept of the proposed “Quantitative Detection Method for Surface Angled Cracks Based on Full-Field Scanning Data” is illustrated in Figure 1. By utilizing the characteristics of signals from different scanning positions in the full-field scanning data, the propagation times of different waveforms are extracted. Combining these with the propagation velocities of Rayleigh and longitudinal waves allows for the calculation of the crack’s width, angle, and length using this method.

The following sections will discuss five key aspects: the characteristics of full-field scanning data, the method for solving the surface crack width, the interaction between ultrasonic waves and near-field cracks, the interaction between ultrasonic waves and far-field cracks, and the determination of the near-field to far-field critical point.

### 2.1. Time-Domain Analysis of Full-Field Scanning Data Characteristics

**Figure 2:**
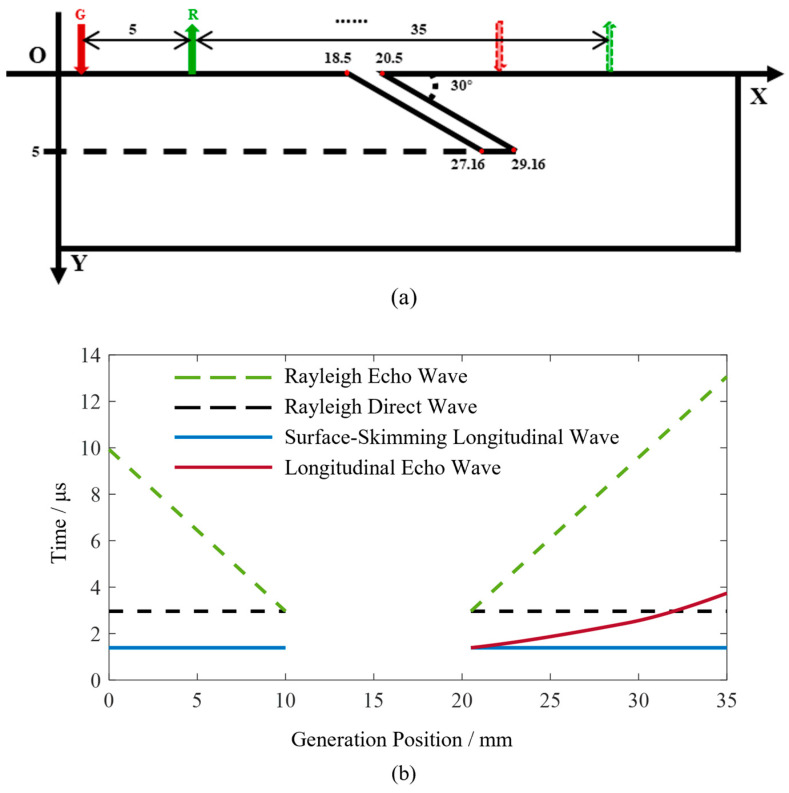
Schematic diagram of surface angled crack full-field scanning and full-field scanning signals for surface angled cracks. (**a**) Schematic diagram of surface angled crack full-field scanning. (**b**) Full-field scanning signals for surface angled cracks.

To further investigate the characteristics of the full-field scanning data, a surface angled crack model, as shown in Figure 2a, is established. The surface angled crack has a lateral width of 2 mm, a vertical depth of 5 mm, and an inclination angle of 30 degrees with respect to the upper surface, slanting to the right. During the detection process, the main interaction occurs between the right edge of the crack and the ultrasonic waves. The length of the right edge is 10 mm. The crack’s upper surface’s starting coordinates are (18.5, 0) and (20.5, 0). The generation point is located at (0, 0) and the starting position of the receiving point is (8.5, 0). The fixed distance between the generation and receiving points is a constant value of 8.5 mm. A scan step of 0.1 mm is set, with a scan range of 35 mm. Both the generation and receiving points move simultaneously to the right during the scanning process. The Rayleigh wave propagation speed is set to 2870 m/s and the longitudinal wave propagation speed is 6120 m/s.

The principle of laser ultrasonic wave generation is based on the interaction between a pulsed laser and the material’s surface. When a high-energy pulsed laser irradiates the surface of the material, the rapid heating causes the surface to instantly expand, generating thermal stress. This thermal stress excites multiple modes of ultrasonic waves, including Rayleigh waves, longitudinal waves, and shear waves, which propagate both on the surface and within the specimen. In this study, the generation point refers to the position where the pulsed laser irradiates the specimen surface, while the receiving point is the location where the interferometer is directed on the specimen surface.

For surface angled cracks, the propagation characteristics of Rayleigh waves can be utilized to calculate the crack opening width and determine the distance between the scanning position and the crack’s starting point. The reflected waves within the longitudinal wave signals contain information that can be used to calculate the inclination angle and length of the crack. Therefore, this paper focuses primarily on the propagation behavior of Rayleigh waves and longitudinal waves. The time-domain signals of Rayleigh waves and longitudinal waves were obtained through numerical calculations, as shown in Figure 2b. The horizontal axis represents the coordinates of the generation point and the vertical axis represents the ultrasonic wave propagation time. In the time-domain signal plot, the dashed line represents the Rayleigh waves, while the solid line represents the longitudinal waves.

The Rayleigh direct wave is the Rayleigh wave that propagates from the generation point to the receiving point along the upper surface. Due to its constant propagation path length, the time of occurrence in the time-domain signal remains unchanged, represented in the figure by the color black. The two dashed lines in the color green represent the Rayleigh wave echoes from the crack, with their propagation paths extending from the generation point to the upper endpoint of the crack and reflecting back to the receiving point. During the scanning process, the detection position gradually approaches the crack, resulting in shorter propagation paths for the Rayleigh wave echoes, which increasingly resemble the Rayleigh direct wave. When the receiving point is located at the upper endpoint on the left edge of the crack, only the Rayleigh direct wave signal is present, with no Rayleigh wave echoes. When both the generation and receiving points are located on the right side of the crack, the Rayleigh wave echoes are collected during the scanning, with propagation times gradually increasing and moving further away from the Rayleigh direct wave.

The longitudinal wave includes a mode that propagates along the upper surface, following the same propagation path as the Rayleigh direct wave. Therefore, its propagation time does not vary with the scanning position. This type of longitudinal wave is known as a surface-skimming wave. When both the generation and receiving points are located on the left side of the crack, the longitudinal wave propagating towards the crack reflects within the specimen, leading to an absence of detectable longitudinal wave echoes from the crack. This is evidenced in the time-domain signal, which shows a lack of longitudinal wave echoes at the 0–10 mm position. As the generation and receiving points continue to scan on the right side of the crack, echoes of the longitudinal wave from the crack can be collected. As the scanning position moves away from the crack, the propagation path length of the longitudinal wave echoes increases, resulting in longer propagation times. The figure shows the distribution of the longitudinal wave echoes from the crack in the time domain as a curve, which will be elaborated on in Section 2.5.

When the generation point is located between 10 mm and 20.5 mm, no ultrasonic signals are detected, which will be explained in detail in Section 2.2.

### 2.2. Method for Determining Surface Crack Width Based on Full-Field Scanning Data

During the full-field scanning of surface angled cracks, the generation and receiving points may initially be positioned on the same side of the crack, then on opposite sides, and finally return to the same side. When the generation and receiving points are on opposite sides of the crack, ultrasonic waves attenuate rapidly in the air, preventing wave propagation through the air gap in the crack. Additionally, when ultrasonic waves propagate along the crack edges, the longer path and energy loss at the corners result in significant attenuation. Therefore, when the generation and receiving points are located on opposite sides of the crack, no effective ultrasonic signals are detected; only some noise is present, as illustrated in the case between 10 mm and 20.5 mm in Figure 2b.

When the distance between the generation and receiving points remains constant, the arrival time of the Rayleigh direct wave is also constant, appearing as a straight line in the B-scan image. However, during full-field scanning, if the generation and receiving points are on opposite sides of the crack, the Rayleigh direct wave cannot be detected. As a result, throughout the scan, the Rayleigh direct wave will exhibit an appear–disappear–appear pattern. The Rayleigh echo wave from the crack will move closer to the Rayleigh direct wave, disappear in between, reappear, and then move away from it as the scanning progresses. By analyzing the positions where the Rayleigh direct wave and echo wave appear, the width of the surface crack can be determined.

When the generation point is between 0 and 10 mm, both the generation and receiving points are on the left side of the crack, allowing the detection of both the Rayleigh direct wave and the Rayleigh echo wave. As the generation point approaches the crack, the Rayleigh echo wave gradually converges with the Rayleigh direct wave. At 10 mm, the receiving point coincides with the crack’s vertex, resulting in the absence of a Rayleigh echo wave; thus, only a single Rayleigh wave is present in the signal. When the generation point is between 10 and 20.5 mm, the generation and receiving points are on opposite sides of the crack, and no ultrasonic signals are detected, indicating a lack of Rayleigh wave signals. At 20.5 mm, the generation point is on the right side of the crack’s vertex, with the receiving point at 29 mm. In this case, both the generation and receiving points are on the same side of the crack, allowing for the generation and detection of ultrasonic signals. As the scanning position moves further away from the crack, the propagation time of the crack’s Rayleigh echo wave gradually increases.

During the full-field scanning process, the distance from the receiving point’s entry into the defect to the generation point’s exit is 10.5 mm, corresponding to the region between 10 and 20.5 mm in the figure. In this experiment, the distance between the generation and receiving points is set to 8.5 mm and the defect width is 2 mm, resulting in a total distance of 10.5 mm. The above analysis can be represented by the following formula:

(1) $$W_{C}=W_{Ri}-d_{GR}$$

where $W_{c}$ is the width of the crack, $W_{Ri}$ is the width of the Rayleigh wave interruption, and $d_{GR}$ is the distance between the generation and receiving point.

In summary, in this model, Rayleigh wave signals are absent in signals starting from the point where the receiving point crosses the left edge of the crack. As the scan progresses to the right, Rayleigh wave signals reappear once the generation point crosses the right edge of the crack. During this process, the length of the missing Rayleigh wave signal, minus the distance between the generation and receiving points, represents the crack opening width.

Based on the above analysis, in full-field scanning, by studying the propagation of Rayleigh direct waves and echoes in the scanning data and determining the location where the Rayleigh wave disappears, the width of the surface crack can be determined by combining the established generation–receiving distance. This method is applicable to all types of surface cracks.

### 2.3. Interaction Between Ultrasonic and Near-Field Crack

**Figure 3:**
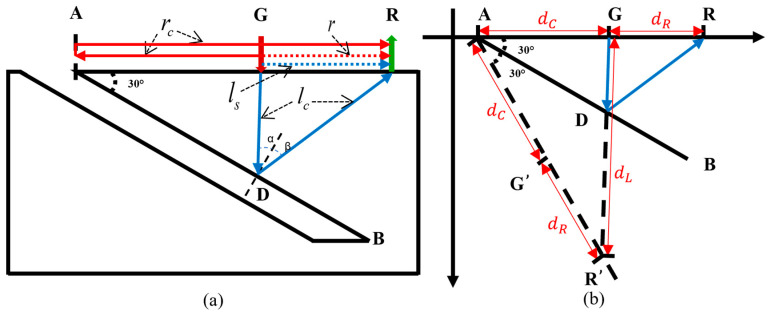
Schematic diagram and abstract geometric model for near-field surface angled crack detection. (**a**) Schematic diagram, (**b**) Abstract geometric model.

To study the interaction between ultrasonic waves and near-field cracks, a geometric model is established, as shown in Figure 3a. AB represents the right edge of an inclined surface crack, where A is the starting point of the crack and B is the endpoint. The generation point G and the receiving point R are positioned on the surface of the specimen near point A on the right side. To ensure that the position of GR is within the near field of the crack, the perpendicular bisector of the GR line segment intersects with AB. Figure 3a illustrates the interaction process between the ultrasonic waves and the near-field crack.

When a pulsed laser is directed at point G, Rayleigh waves are generated and propagate along the upper surface, and the receiving point R will receive two Rayleigh wave signals: one is the direct Rayleigh wave r that propagates from point G to R, and the other is the Rayleigh wave echo $r_{c}$ that reflects from crack upper endpoint A back to R. Longitudinal waves generated by the pulsed laser propagate into the specimen. The longitudinal waves traveling along the upper surface to the receiving point are referred to as the surface-skimming longitudinal wave $l_{s}$. When the longitudinal wave reaches point D on the crack, a normal line to AB is drawn at point D. When α=β, the longitudinal wave reflected by the crack reaches the receiving point R, and the path of the crack’s longitudinal wave echo is G-D-R, denoted as $l_{c}$.

In studying the interaction between ultrasound and a near-field crack, an abstract geometric model is shown in Figure 3b. The propagation speed of the Rayleigh wave in the material is $v_{r}$, and the propagation speed of the longitudinal wave is $v_{l}$. An auxiliary line is drawn as a dashed line, symmetric to the upper surface with respect to the axis AB. Extending GD intersects the auxiliary line at $R^{\prime }$. According to the law of reflection, $G^{\prime }$ is the symmetric point of G, $R^{\prime }$ is the symmetric point of R, $∠GAB=∠G^{\prime }AB$, and $AR=AR^{\prime }$, thus $DR=DR^{\prime }$. In $△AGR^{\prime }$, the length of AG is $(t_{rc}-t_{r})/2\times v_{r}$, the length of $AR^{\prime }$ is $(t_{r}+t_{rc})/2\times v_{r}$, and the length of $GR^{\prime }$ is $t_{lc}\times v_{l}$. Using the cosine law, the angles of the triangle can be calculated from the side lengths. Since $∠GAB=\frac{1}{2}∠GAR^{\prime }$, ∠GAB can be determined using the following formula:

(2) $$\left\{ \begin{array}{l} d_{C}=\frac{\left(t_{rc}-t_{r}\right)}{2}\cdot v_{r}\\ d_{R}=t_{r}\cdot v_{r}\\ d_{L}=t_{lc}\cdot v_{l}\\ ∠GAB=\frac{1}{2}\arccos\left(\frac{{d_{C}}^{2}+{\left(d_{C}+d_{R}\right)}^{2}-{d_{L}}^{2}}{2d_{C}\left(d_{C}+d_{R}\right)}\right) \end{array}\right.$$

From the derivation, it is evident that in near-field crack detection, by extracting the propagation times of the relevant waveforms from the ultrasonic signals, the angle between the crack and the upper surface can be determined using the formula provided above.

### 2.4. Interaction Between Ultrasonic and Far-Field Crack

**Figure 4:**
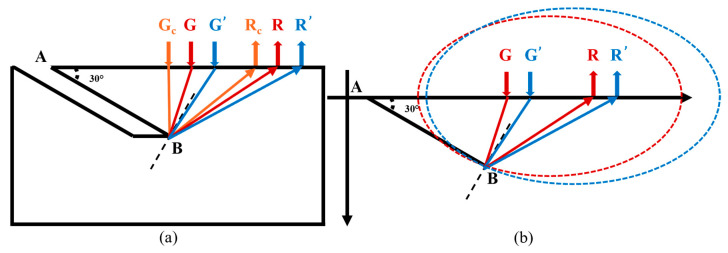
Schematic diagram and abstract geometric model for far-field surface angled crack detection. (**a**) Schematic diagram, (**b**) Abstract geometric model.

As the scanning position moves farther from the crack, as shown in Figure 4a, we assume that points $G_{c}$ and $R_{c}$ are the critical locations for near-field crack detection. At point $G_{c}$, the reflection of the longitudinal wave occurs precisely at the crack’s endpoint B, satisfying the law of reflection. In this configuration, the perpendicular line drawn from point B along the crack line AB bisects the angle $∠G_{c}BR_{c}$.

As the scanning continues to the right, the longitudinal wave generated from point G reaches point B and reflects in all directions from B, acting as a point source. At this stage, the propagation path of the longitudinal wave echo from the crack that reaches point R is G-B-R. In this scenario, it becomes clear that the perpendicular line from B cannot bisect ∠GBR, meaning the model in Figure 3b is no longer applicable, and thus, formula 2 cannot be used.

As the scanning progresses (as shown in Figure 4a with points $G^{\prime }$ and $R^{\prime }$), all longitudinal wave echoes in the ultrasonic signal originate from point B. Therefore, starting from positions $G_{c}$ and $R_{c}$, the reflection points on the crack remain fixed at B, unlike in near-field detection, where the reflection points vary with the scanning position. Consequently, beyond $G_{c}$ and $R_{c}$, the detection transitions into the far-field region of the crack.

For the far-field detection scenario depicted in Figure 4a, an abstract geometric model is presented in Figure 4b. In a single measurement, let points G and R be two fixed points, and point B be an unknown point located outside these two points. Given the known length of the G-B-R path, an ellipse is constructed with G and R as the foci and G-B-R as the major axis. According to the definition of an ellipse, point B lies on this ellipse. The equation of the ellipse is as follows:

(3) $$\left\{ \begin{array}{l} a=\frac{GB+BR}{2}\\ c=\frac{GR}{2}\\ b=\sqrt{{\left(a\right)}^{2}-{\left(c\right)}^{2}}\\ \frac{x^{2}}{a^{2}}+\frac{y^{2}}{b^{2}}=1 \end{array}\right.$$

Although a single measurement establishes that point B lies on the ellipse, it does not provide the exact position of B. When scanning reaches the position $G^{\prime }R^{\prime }$, an ellipse constructed with $G^{\prime }$ and $R^{\prime }$ as the foci will also pass through point B. Therefore, the intersection of the two ellipses—one with foci G and R, and the other with foci $G^{\prime }$ and $R^{\prime }$—can precisely determine the location of B, as illustrated in Figure 4b [20].

In multiple far-field crack detections, by extracting the propagation times of the target waveforms from the signals and using these times to draw multiple ellipses according to the formula, the intersection points of these ellipses can be used to determine the crack’s endpoint. By combining the endpoint with the known starting point of the crack, the length of the crack can be accurately determined.

In practical detection, system errors, noise, and the significant attenuation of longitudinal waves in far-field signals can lead to inaccuracies in the extracted ultrasonic propagation times. When these times are used to draw ellipses, the ellipses may not intersect at a single point. Theoretically, any two ellipses should intersect at the crack’s endpoint. Therefore, by examining all ellipses and finding the intersection points of any two ellipses, the centroid of these intersection points can be considered the crack’s endpoint.

To simplify the calculation process, this paper proposes a method that uses intersection points of adjacent ellipses to determine the target point. By extracting the ultrasonic propagation times from n far-field signals and drawing n ellipses according to the formula, there will be n−1 intersection points (excluding symmetric points) between the adjacent ellipses. The centroid of these n−1 points can be regarded as the crack’s endpoint.

When the extracted ultrasonic propagation times are inaccurate, adjacent ellipses may fail to intersect, resulting in a large ellipse encompassing a smaller one. Although, theoretically, the intersection points of any two ellipses should indicate the defect’s endpoint, having enough intersections allows the centroid of these points to accurately approximate the crack’s endpoint. Therefore, cases where adjacent ellipses do not intersect can be disregarded, and the centroid of the intersection points from the ellipses that do intersect can be used to approximate the crack’s endpoint.

### 2.5. Determination of Near-Field and Far-Field Critical Positions

The previous two sections discussed the interaction between ultrasonics and cracks in both the near-field and far-field regions, enabling the determination of the crack’s inclination angle and length. However, in practical B-scan inspections, the echo signals are often weak, making it difficult to directly identify the near-field and far-field critical positions from the signals. As shown in the longitudinal wave echoes from the crack in Figure 2b, it can be observed that the distribution of the longitudinal wave echoes varies around the 30 mm position; however, the exact location cannot be determined.

**Figure 5:**
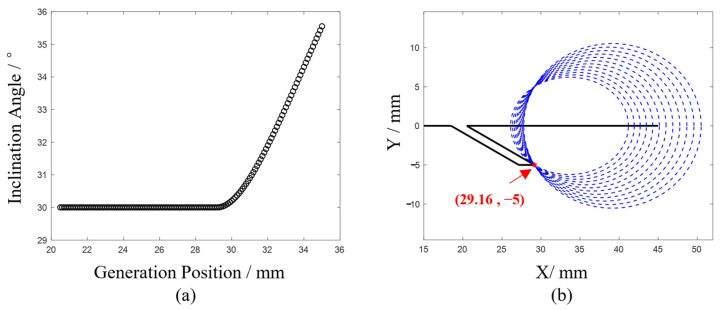
(**a**) Diagram between generation position and calculated inclination angle; (**b**) Elliptical positioning algorithm for locating crack endpoint in far-field detection. (**a**) Calculated inclination angle. (**b**) Result of far-field detection.

To further investigate the critical point, the results from the scanning process were substituted into Formula (2) for calculating the crack inclination angle, as shown in Figure 5a. The inclination angle begins to increase linearly from the 29.4 mm position. Geometrically, this can be understood as solving for an angle given the lengths of the three sides. During the fixed-step scanning process, the distance RA (from the receiving point to the crack’s starting point) changes linearly. After transitioning from near-field to far-field detection, the reflection point consistently becomes B, and the growth rate of G-B-R exceeds that of RA. Consequently, when calculating the crack angle, the angle corresponding to G-B-R increases at a faster rate, which aligns with the results shown in Figure 5a. Thus, the 29.4 mm position is identified as the critical point marking the transition from near-field to far-field detection.

From the above analysis, it can be concluded that when the generation point is located beyond 29.5 mm, the conditions for far-field detection are satisfied. Using an interval of 0.5 mm, the ultrasonic wave propagation times at the corresponding positions are substituted into Formula (3), resulting in 12 sets of elliptical curves, as shown in Figure 5b. As illustrated in the figure, ellipses constructed with the generation and receiving points as foci and the longitudinal wave echo length as the major axis intersect at the point (29.16, −5). This calculated intersection aligns with the crack model, demonstrating that the elliptical positioning algorithm effectively resolves the defect endpoint problem.

In practical applications, substituting the full-field scanning data into Formula (2) and analyzing the variations in the crack inclination angles allows for the determination of the critical point between near-field and far-field detection. Different algorithms are employed for near-field and far-field data to determine the crack’s inclination angle and endpoint, which subsequently facilitates the calculation of the crack length.

Section 2.1, Section 2.2, Section 2.3, Section 2.4 and Section 2.5 study the time-domain characteristics of the full-field scanning data, analyzing the interaction patterns between ultrasonic waves and cracks at different scanning positions. Based on the propagation characteristics of different waveforms, the width of the surface crack, the angle with respect to the upper surface, and the position of the crack endpoints are determined, leading to the calculation of the crack length. The proposed “Quantitative Detection Method for Surface-Angled Cracks Based on Full-field Scanning Data” analyzes the extensive information within the full-field scanning data to ultimately output the width, angle, and length of the surface angled cracks, achieving the quantitative detection of surface angled cracks.

## 3. Experiment and Results

To validate the accuracy of the “Quantitative Detection Method for Surface Angled Cracks Based on Full-Field Scanning Data”, experiments were conducted to assess its effectiveness on surface cracks with varying inclination angles. The experimental setup and procedures are detailed below.

### 3.1. Experimental Equipment

**Figure 6:**
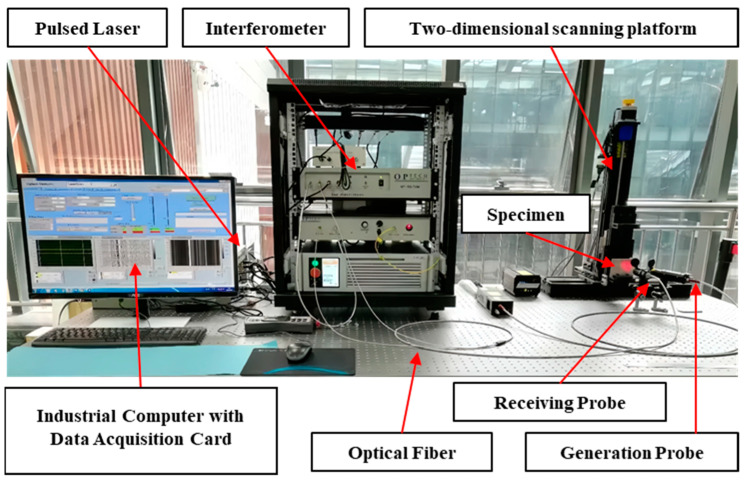
Laser ultrasonic testing system.

The laser ultrasonic testing system employed in this experiment is operated via an industrial computer, comprising both a control unit and a data acquisition unit. After setting the scanning parameters on the industrial computer, control commands are sent to the motion platform, which moves the sample to perform the scan. Simultaneously, the system coordinates the pulsed laser generation, beam splitter, and receiver to capture the surface vibration signals of the sample. The ultrasonic signals collected by the data acquisition board are then transmitted to the computer for storage and further analysis. The laser ultrasonic testing system is shown in Figure 6.

The key components and their specifications are as follows:

(1) Scanning Platform:
  ○ Type: two-dimensional scanning platform.
  ○ Movement Step: 0.1 mm.
  ○ Movement Range: 25 mm.
(2) Pulsed Laser:
  ○ Type: Q-switched Nd:laser.
  ○ Wavelength: 1064 nm.
  ○ Energy: 42.3 mJ/pulse.
  ○ Pulse Width: 8 ns.
(3) Interferometer:
  ○ Type: dual-wavelength interferometer.
  ○ Wavelength: 532 nm.
  ○ Sensitivity: 4×10−7nm/(W/Hz)12.
  ○ Laser Power: 2 W.
(4) Data Acquisition Card:
  ○ Model: PCI-5114.
  ○ Bandwidth: 125 MHz.
  ○ Sampling Frequency: 250 MS/s.
(5) Probes:
  ○ Type: both generation and receiving probes.
  ○ Aperture: 25 mm.
  ○ Spot Size: approximately 1 mm.
  ○ Focal Length: 200 mm.

### 3.2. Experimental Specimen and Setup

Research on crack defects in blades [21,22] and bearings [23] reveals that most surface angled cracks form an angle of 45° to 90° with the upper surface, while some can have an inclination of up to 30° in specific regions. The crack opening width is typically under 1 mm, but may reach 2 mm in thicker areas, potentially leading to rapid fractures. Crack opening lengths vary depending on the component, material, and location. To verify the accuracy of the proposed detection method, four experimental specimens have been designed to simulate real defects.

**Figure 7:**
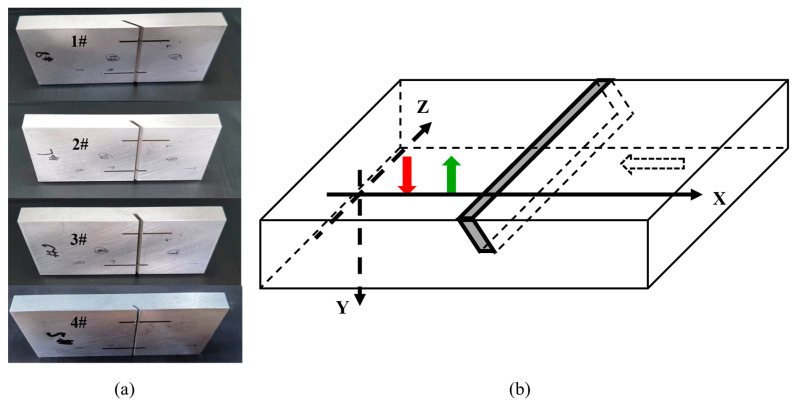
Specimens and schematic diagram of experiments. (**a**) Specimens, (**b**) Schematic diagram of experiments.

Four ER2319 aluminum alloy specimens were prepared, each with dimensions of $100\times 10\times 45\;mm^{3}$. The Rayleigh wave propagation speed in these specimens is 2870 m/s, and the longitudinal wave propagation speed is 6120 m/s. Surface angled cracks were prefabricated on the top surface of each specimen, as shown in Figure 7a. The parameters of the four surface angled cracks are consistent with those listed in Table 1.

The experimental setup is as follows: The distance between the generation point and the receiving point on the specimen is fixed at 8.5 mm. The crack is oriented towards the right. The initial scanning position is located to the left of the crack, and the specimen is moved to the left during scanning. The scan step is 0.1 mm, covering a total scan length of 25 mm, resulting in the collection of 250 sets of signals. Each scanning position is sampled 32 times, and time-domain averaging is applied to reduce random errors. The sampling frequency is 125 MHz, and each signal is recorded over a duration of 10 μs. A schematic of the experiments is shown in Figure 7b.

### 3.3. Results

**Figure 8:**
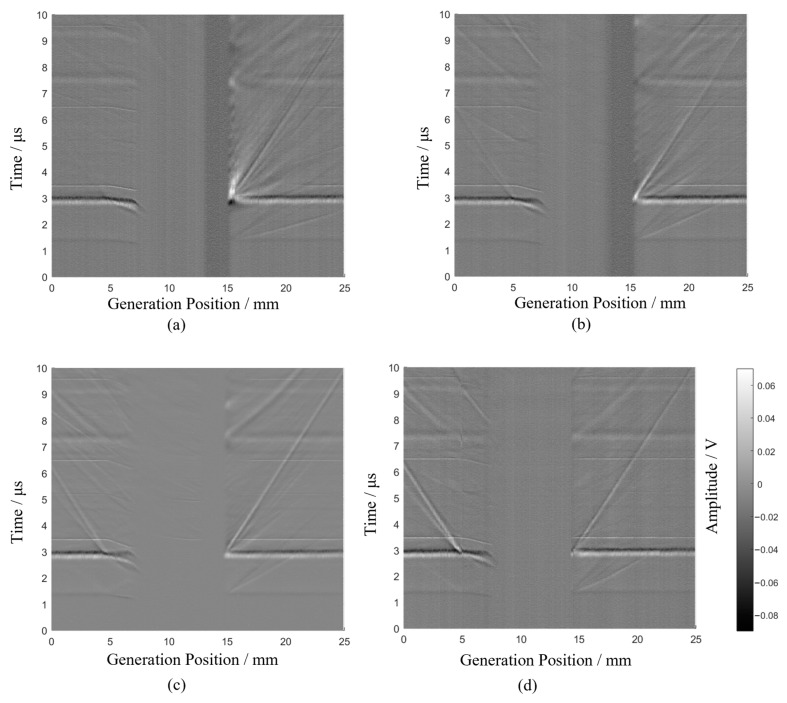
Raw full-field b-scan signals for surface angled cracks with different inclinations. (**a**) Experiment 1#, (**b**) Experiment 2#, (**c**) Experiment 3#, (**d**) Experiment 4#.

The raw data obtained from the four experiments are shown in Figure 8, where the horizontal axis represents the position of the generation point and the vertical axis represents time.

#### 3.3.1. Signal Processing

In the proposed detection method, significant calculations are based on the propagation times of Rayleigh waves and longitudinal waves. To accurately extract the ultrasonic propagation time from the raw signals, a series of time windows are defined in the B-scan signals according to the propagation characteristics of different modal ultrasonic waves. The midpoint between the maximum and minimum amplitudes within each time window is used as the ultrasonic propagation time. For the overlapping portions of longitudinal and Rayleigh waves, the longitudinal wave propagation time is extracted from the raw signal by subtracting the defect-free reference signal within the specified time window.

Taking the third experiment as an example, this group includes overlapping portions of longitudinal and surface waves, representing the most complex scenario in signal processing.

**Figure 9:**
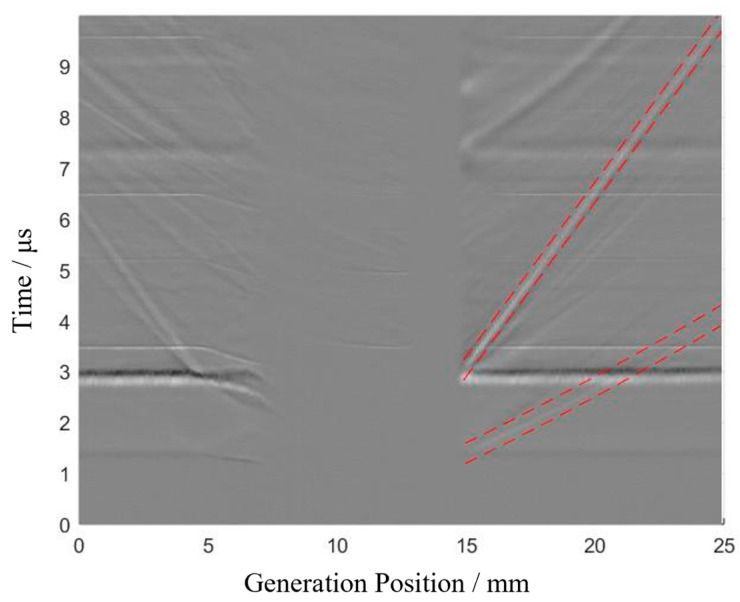
Raw signal for experiment 3#. (The red dashed line represents the time window).

For the Rayleigh wave defect echo, as shown in Figure 9, it extends linearly from the position at 15 mm and 3 μs to 25 mm and 10 μs. Therefore, the propagation path of the Rayleigh wave defect echo is fitted accordingly. Based on this path, two red dashed lines are set at ±0.2 μs to define the time window, within which the propagation time of the Rayleigh wave defect echo is extracted following the previously mentioned method.

For the longitudinal wave defect echo, since the initial part of the echo path is linear and the latter part forms a curve, giving it an overall parabolic shape, ten points are manually selected to fit the propagation path of the longitudinal wave defect echo. Based on this parabolic path, two red dashed lines at ±0.2 μs define the time window, and the propagation time of the longitudinal wave defect echo is then extracted within this window following the aforementioned method.

**Figure 10:**
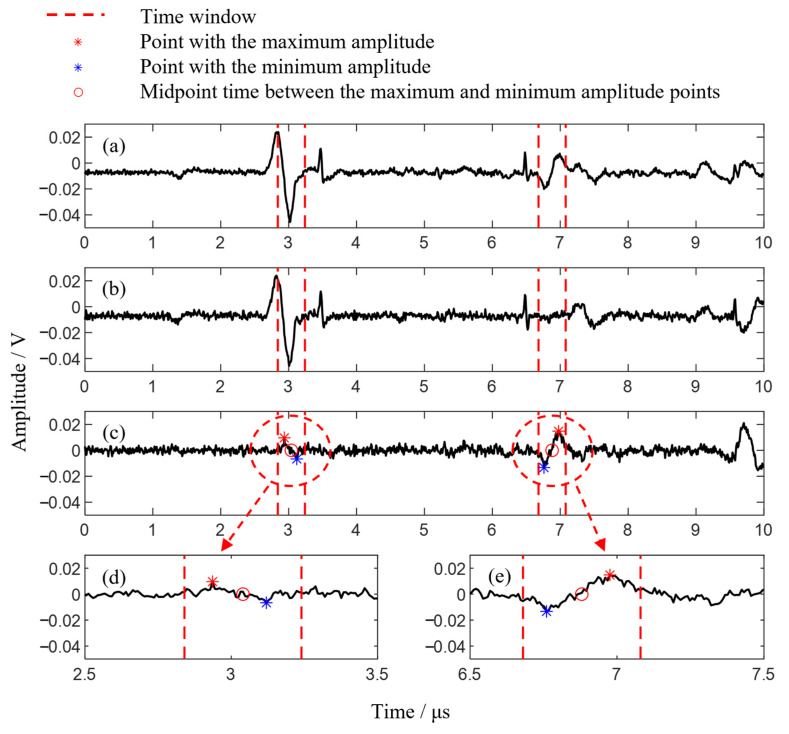
Extraction of ultrasonic propagation time from the signal: (**a**) Raw signal; (**b**) Defect-free reference signal; (**c**) Result of subtracting the reference signal from the raw signal; (**d**) Extraction of the longitudinal wave defect echo; (**e**) Extraction of the Rayleigh wave defect echo.

Taking the signal at 20.6 mm as an example, the raw signal is shown in Figure 10a, where the defect echo of the Rayleigh wave is clearly identifiable. However, the defect echo of the longitudinal wave is superimposed with the direct arrival of the Rayleigh wave, making it difficult to accurately determine the propagation time of the longitudinal wave defect echo from the signal. Figure 10b presents the defect-free reference signal, while Figure 10c shows the difference between the raw and reference signals. Here, the direct Rayleigh wave and the longitudinal wave’s backwall echo are suppressed, enhancing the amplitude of the longitudinal wave defect echo at 3 μs. Figure 10d,e provide an enlarged view of the time-windowed portion of Figure 10c. In Figure 10d, the red asterisk indicates the point with the maximum amplitude within the time window, the blue asterisk marks the point with the minimum amplitude, and the red circle represents the midpoint time between the maximum and minimum amplitudes. This midpoint time is taken as the propagation time of the defect echo and is then used in the proposed method for subsequent calculations.

#### 3.3.2. Calculation of Crack Width

The signal with the highest amplitude appears at 3 µs. When the generation point is between 0–7 mm and 15–25 mm, the propagation time of this waveform remains unchanged with the scanning position. This indicates that this signal is the Rayleigh direct wave, as inferred from the Rayleigh wave speed and scanning parameters.

During the scan, there are positions where the generation and receiving points are on opposite sides of the defect. As analyzed in Section 2.2, no ultrasound signal is detected at these positions, resulting in random noise between the 7 mm and 15 mm positions in Figure 8.

When both the generation and receiving points are on the same side of the crack, the Rayleigh wave echo from the crack is observable in the signal, as shown in Figure 2b. As scanning progresses, the Rayleigh echo wave approaches the Rayleigh direct wave and disappears between 5 mm and 15 mm, as depicted in Figure 8. Beyond 15 mm, the Rayleigh echo wave reappears and moves away from the Rayleigh direct wave.

Based on the analysis in Section 2.2, the point where the Rayleigh echo wave overlaps with the Rayleigh direct wave is identified. Using the distance between the generation and receiving points, the crack width is calculated using Formula 1. The calculated crack widths from the four experiments are 2.1 mm, 1.9 mm, 1.9 mm, and 1.0mm, respectively, which are consistent with the actual crack widths.

#### 3.3.3. Calculation of Crack Angle

To accurately determine the crack’s inclination angle and length, it is observed that no longitudinal wave echoes from the crack are present in the first 150 signals of each experiment. From the 150th signal onwards, the scan transitions from near-field to far-field detection. Consequently, the last 100 signals from each experiment are analyzed using the methodology outlined in Section 2.

**Figure 11:**
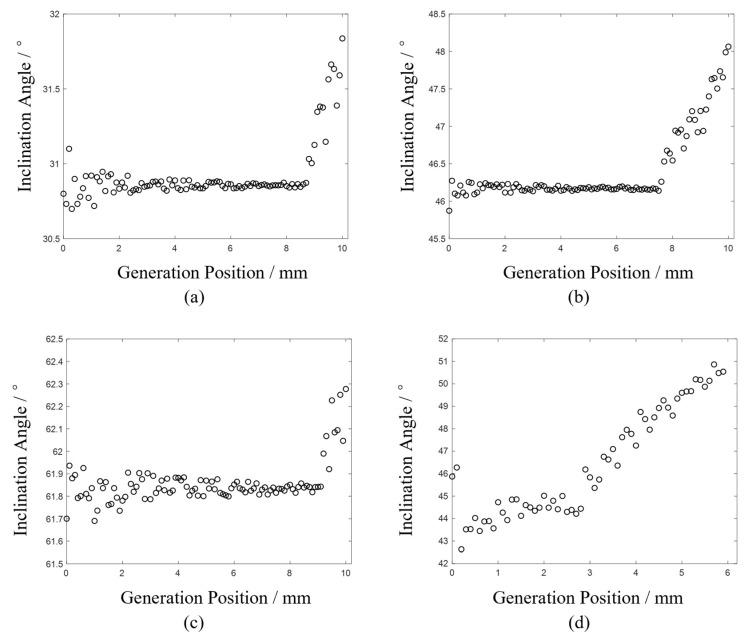
Diagram between generation position and calculated inclination angle. (**a**) Experiment 1#, (**b**) Experiment 2#, (**c**) Experiment 3#, (**d**) Experiment 4#.

In the analysis, a time window is applied to both the Rayleigh wave and longitudinal wave echoes. The midpoint between the maximum and minimum amplitudes within this window is selected as the echo moment. Using the Rayleigh and longitudinal wave echoes from the last 100 signals of each experiment, the crack inclination angles at various scan positions are calculated and the results are presented in Figure 11.

The trends observed in the results of the four experiments align with those depicted in Figure 5a. Initially, the calculated angles exhibit some fluctuation during the scan. As the scan progresses, the results stabilize at a constant value, but once the scan surpasses the critical point, the calculated angles increase rapidly.

When the scanning position is near the crack, the time difference between the longitudinal wave echo from the crack and the surface-skimming longitudinal wave is minimal. Consequently, extracting the longitudinal echo wave from the crack within the specified time window can be significantly influenced by the surface-skimming longitudinal wave. This interference contributes to larger errors in the calculation, which explains the pronounced fluctuations observed in the first 20 data points of Figure 11a,c.

The point at which the angle begins to increase in Figure 11 indicates the transition from near-field to far-field detection. For example, in Figure 11a, the first 87 data points represent near-field detection results, and their average value reflects the inclination angle of the crack. Similarly, Figure 11b shows the near-field detection results for the first 75 data points and Figure 11c for the first 92 data points. In Figure 11d, the first 30 data points represent the near-field detection results, and their average value reflects the inclination angle of the crack. Averaging the near-field detection angles from the four experiments yields values of 30.85°, 46.17°, 61.83°, and 44.42°, respectively, which are slightly larger than the actual crack inclination angles.

This discrepancy is attributed to hardware-related asynchrony between the generation laser, movement module, and interferometer during simultaneous operation. This asynchrony causes the measured longitudinal wave echo time to be slightly longer than the actual time, leading to overestimated values in the calculations.

#### 3.3.4. Calculation of Crack Length

**Figure 12:**
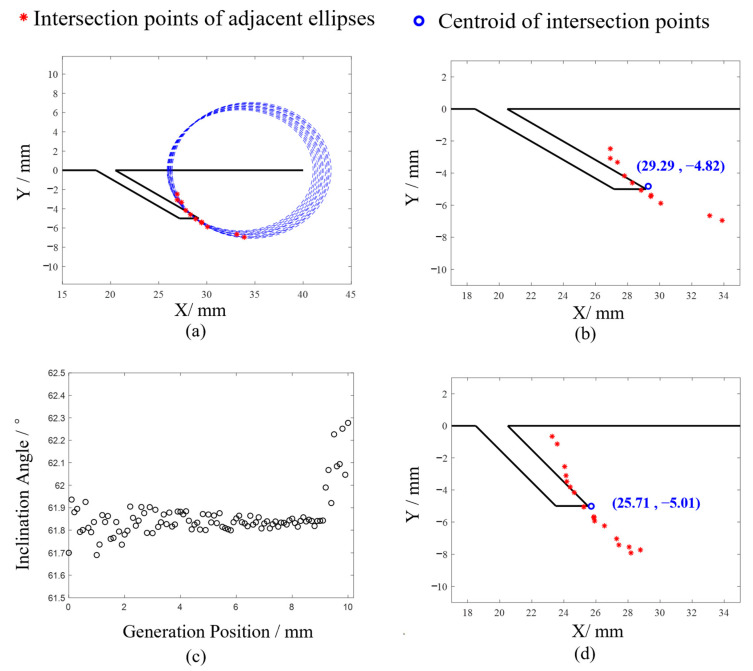

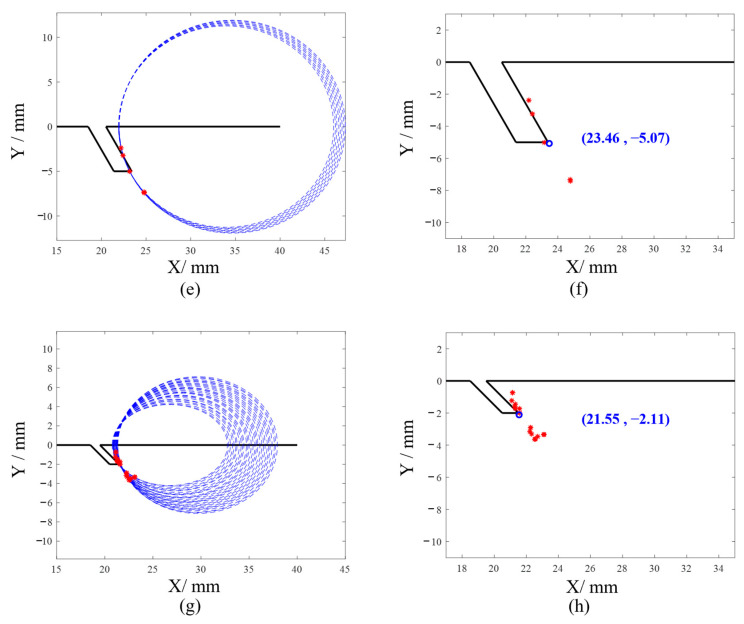
Elliptical positioning algorithm for locating endpoints of far-field surface angled cracks. (**a**) Experiment 1#, (**b**) Experiment 1#, (**c**) Experiment 2#, (**d**) Experiment 2#, (**e**) Experiment 3#, (**f**) Experiment 3#, (**g**) Experiment 4#, (**h**) Experiment 4#.

After the critical point, the far-field detection data are processed using the elliptical positioning algorithm, with the results presented in Figure 12.

In Figure 12a,c,e,g, the results are consistent with those shown in Figure 5b, where the intersection points of the adjacent ellipses are distributed around the crack tip. However, in Figure 12, discrepancies are observed as some ellipses do not intersect at a single point. This deviation from the theoretical calculations results is attributed to errors in extracting the ultrasound propagation times from the raw data, which are then magnified during calculations. Consequently, some adjacent ellipses fail to intersect. For instance, in Figure 12d, there are, theoretically, 26 ellipses, which should produce 25 intersection points. However, due to calculation errors, 7 couples of ellipses do not intersect, and only 18 intersection points are observed.

Figure 12b,d,f,h illustrate the relative positions of the intersection points of adjacent ellipses with respect to the crack. The central coordinates of the intersection points are (29.29, −4.82), (25.71, −5.01), (23.46, −5.07), and (21.55, −2.11) which are considered as the crack tips. Using these calculated crack tip positions along with the crack’s starting point, the crack lengths are determined to be 10.02 mm, 7.23 mm, 5.87 mm, and 2.83 mm, respectively, which align with the actual crack parameters.

#### 3.3.5. Summary of Experimental Results

The summary of the experimental results is shown in Table 2. The detection errors in all the experiments are within 5%, demonstrating that the proposed “Quantitative Detection Method for Surface Angled Cracks Based on Full-field Scanning Data” is accurate in determining the width, angle, and length of surface angled cracks. This validates the feasibility of the method for the quantitative detection of surface angled cracks.

## 4. Conclusions

This paper conducts an in-depth analysis of laser ultrasonic full-field scanning signals and proposes a ‘Quantitative Detection Method for Surface Angled Cracks Based on Full-field Scanning Data’. The interaction patterns between different waveforms and cracks are investigated through theoretical calculations. The crack width is determined using Rayleigh waves, and a method for identifying the critical point of transition from near-field to far-field detection is introduced. Different formulas are applied to obtain the inclination angle and endpoints of the crack based on various detection positions, and the feasibility of the method is validated through experiments, resulting in measurements of the crack width, inclination angle, and length with errors all within 5%.

By employing this method for the quantitative detection of surface angled cracks in critical components, a comprehensive assessment can be made, aiding in estimating the component service life and proactively mitigating operational risks. Additionally, this method can output multiple crack parameters in a single detection, making it an efficient approach. It shows promising application potential in fields such as aircraft engine blade inspections and gear inspections.

However, this method also has certain limitations. It performs well for linear or upwardly protruding defects, but for recessed defects, multiple reflection points may occur during a single detection, making it difficult to determine the inclination angle of the cracks in subsequent calculations.

## Figures and Tables

**Table 1:** Parameters of surface angled cracks in different specimens.

Specimen Number	1#	2#	3#	4#
Crack Inclination Angle/°	30	45	60	45
Surface Opening Width/mm	2.00	2.00	2.00	1.00
Vertical Depth/mm	5.00	5.00	5.00	2.00
Horizontal Width of Right Inclined Edge/mm	8.70	5.00	2.90	2.00
Length of Right Inclined Edge/mm	10.00	7.07	5.78	2.83

**Table 2:** Summary of Experimental Results.

Object	1#	2#	3#	4#
Crack inclination Angle/°	30	45	60	45
Calculated Angle/°	30.85	46.17	61.83	44.42
**Angle Error**	**2.83%**	**2.60%**	**3.05%**	**1.29%**
Crack Tip	29.16, −5.00	25.50, −5.00	23.39, −5.00	21.50, −2.00
Calculated Tip	29.29, −4.82	25.71, −5.01	23.46, −5.07	21.55, −2.11
**Tip Distance/mm**	**0.22**	**0.21**	**0.10**	**0.12**
Crack length/mm	10.00	7.07	5.78	2.83
Calculated length/mm	10.02	7.23	5.87	2.94
**Length Error**	**2.00%**	**2.26%**	**1.56%**	**3.89%**
Crack Width/mm	2.00	2.00	2.00	1.00
Calculated Width/mm	2.10	1.90	1.90	1.00
**Width Error**	**5%**	**5%**	**5%**	**0**

## Data Availability

The data presented in this study are available on request from the corresponding author. The data are not publicly available due to confidentiality.
